# Creating virtual communities of practice for ambulance paramedics: a qualitative evaluation of the use of Project ECHO in end-of-life care

**DOI:** 10.29045/14784726.2022.12.7.3.51

**Published:** 2022-12-01

**Authors:** Andrew Hodge, Jane Manson, Laura McTague, Sam Kyeremateng, Paul Taylor

**Affiliations:** Yorkshire Ambulance Service NHS Trust ORCID iD: https://orcid.org/0000-0002-2632-2249; St. Luke’s Hospice Sheffield; St. Luke’s Hospice Sheffield; St. Luke’s Hospice Sheffield; St. Luke’s Hospice Sheffield

**Keywords:** education, palliative care, paramedic

## Abstract

**Introduction:**

Ambulance services play a key role in the recognition and care of patients nearing their end of life, yet are expected to recognise and manage these complex presentations often with limited education. Paramedics operate across large geographical areas, meaning education delivery is challenging. Yorkshire Ambulance Service implemented Project Extension for Community Healthcare Outcomes (ECHO), which is the creation of virtual communities of practice to address this problem and increase access to specialist supervision, education and sharing of practice. We undertook a service evaluation of the programme and interviewed paramedics about their experiences with ECHO.

**Methods:**

Semi-structured interviews were conducted with eight ambulance clinicians who took part in the end-of-life care (EoLC) ECHO programme. Thematic analysis and coding was undertaken to identify and develop the emerging themes.

**Results:**

This study identified three key themes: programme structure, factors influencing engagement and professional impact. The provision of a virtual community of practice through Project ECHO was a unique and highly valued experience, which was accessible and allowed for networking, peer support and sharing of practice. The concept of a ripple effect was reported in disseminating learning across the wider team.

**Conclusion:**

The development of virtual communities of practice as a novel educational intervention has the potential to transform clinical supervision and ongoing education for ambulance clinicians who are often isolated by the nature of ambulance services that cover large regions.

## Introduction

High-quality end-of-life care (EoLC) demands the provision of services that are multi-agency, co-ordinated, responsive and able to communicate the patient’s wishes to all those involved in their care. Clinician knowledge and skill are key components that underpin successful service delivery, yet with an aging population more care now falls to the generalist clinician in collaboration with specialist palliative care teams (National Palliative and End of Life Care Partnership, 2015).

The challenges in EoLC relate to the complexities in recognising dying, the prognostic uncertainty partly associated with the wide variety of clinical signs and of the dying process itself (Domeisen Benedetti et al., 2013; Kennedy et al., 2014; Kumagai et al., 2012) and confidence in communication (Anselm et al., 2005), all of which may affect patient experience. Studies suggest a correlation between increased clinical confidence and improved communication, advance care planning, service co-ordination and the availability of anticipatory medicines (Faull et al., 2013; Seymour et al., 2010; Thompson et al., 2003).

Often identified as a key enabler, education is found in most policy drivers and guidance (National End of Life Care Programme et al., 2009; National Institute for Health and Care Excellence, 2015), and is also recognised by clinicians themselves as essential to developing practice (Aslakson et al., 2012; Gutierrez, 2012) and improving patient experience.

For the paramedic profession, a number of challenges exist unique to pre-hospital and EoLC in what is acknowledged as a complex and infrequently encountered clinical situation (Pettifer & Bronnert, 2013). The shift in expectations placed upon the profession now asks that the paramedic be competent in the management of urgent and complex conditions in the community, with a broader scope than the traditional training of life-saving skills (Brady, 2014). Moreover, EoLC is notoriously challenging for paramedics, who must differentiate the dying patient requiring life-saving interventions from those requiring palliative care at their end of life (Hoare et al., 2018).

Paramedics are expected to respond to 999 calls without any prior knowledge of the patient, commonly under stressful circumstances, and make rapid assessments often without access to advance care plans. Challenges in relation to making best-interest decisions, communication, assessing capacity and making non-conveyance decisions have been linked to education provision (Kirk et al., 2017; Murphy-Jones & Timmons, 2016; Oosterwold et al., 2018). Personal impact from low confidence in holding difficult conversations is associated with stress and well-being issues for those clinicians involved (Douglas et al., 2013).

National ambulance guidance and reports highlight collaboration, support and education as essential elements to service improvement (Association of Ambulance Chief Executives, 2012; Public Health England, 2015). The provision of voluntary continued education and development for paramedics presents challenges associated with the large geographical distances covered by ambulance services. This may result in inequality of access and inconsistent educational opportunities for those clinicians who live furthest from the educational site.

Project Extension for Community Healthcare Outcomes (ECHO) aims to address these geographical challenges using video-conferencing software to allow individuals to dial in to a centralised ‘hub’. Experts are able to remotely educate and supervise, while participants bring case studies to share practice and learn from both presenting experts and peers. ECHO aims to de-monopolise specialist knowledge and share practice among the wider team, requires fidelity to a specific facilitation methodology and has potential in a wide variety of disciplines (University of New Mexico [UNM], 2018). Project ECHO is underpinned by social cognitive theory, situational learning theory and communities of practice to explain the learning and behavioural change principles (Arora et al., 2010).

Since its development in 2003, published literature has demonstrated that developing virtual communities of practice benefits clinician utility, self-reported measures of competence and patient outcomes (Arora et al., 2011; De Witt Jansen et al., 2018; Farris et al., 2017; Mazurek et al., 2017). One systematic review has concluded that Project ECHO is an effective and potentially cost-saving model while acknowledging that more studies are required to demonstrate outcome measures (Zhou et al., 2016).

In the United Kingdom, since 2018, Yorkshire Ambulance Service and St. Luke’s Hospice Sheffield have been delivering an EoLC ECHO programme for paramedics and other ambulance clinicians. Each programme runs for five months and consists of a monthly session following a curriculum developed by the participants themselves. Guest expert speakers are invited to deliver short didactic presentations relevant to the curriculum, followed by two anonymised case studies by the group allowing for learning and sharing of practice across the virtual community. Programme evaluations have demonstrated improvements in self-reported measures of competence and confidence (McTague et al., 2019).

Despite Project ECHO now being delivered in more than 30 countries (UNM, 2018), this was the first funded network in the world for an ambulance service. This evaluation aims to understand the potential for Project ECHO in developing an EoLC community of practice for ambulance clinicians. The objectives were to explore participants’ experiences of Project ECHO, and to understand the benefits and challenges of participating in this programme and the perceived impact on clinical practice.

## Methods

All 30 participants who joined the ECHO programme were invited by email to take part in the evaluation, with eight agreeing to be interviewed and providing consent. The interviews were designed and conducted by the lead author who was part of the ECHO delivery programme. The questions and themes of the interviews were informed from a review of those used in previous studies examining ECHO programmes. A draft of the interview schedule is presented in Table 1.

**Table 1.** Interview schedule.

Component	Questions
Background	Years in the NHS Years in the Ambulance Service Qualifications Age Work experience Current role
Questions related to ECHO	Tell me about your experience of participating in the ECHO programme. What were your reasons for participating in the ECHO programme? What were the benefits and challenges of taking part in the ECHO programme? Did the curriculum, including the case studies and didactic materials, meet your learning needs? If so, in what way? If not, why not? What are your thoughts on the range of didactic trainers, case studies and learning with participants from across the region from different professional backgrounds? Has participating in the ECHO programme had any impact on your clinical practice? If so, in what way? If not, why not? What are your thoughts on the future of the ECHO programme within Yorkshire Ambulance Service? Do you see a need for continuing ECHO community of practice programmes in other subjects?
Implementation facilitators/barriers	Factors that make ECHO successful. Factors that made ECHO challenging to participate in. Curriculum relevance/development. Relationships – clinical role modelling and co-lead input from team. Programme format.

ECHO: Extension for Community Healthcare Outcomes.

Zoom video-conferencing software was used to conduct the 1.5-hour semi-structured interview and record the data. All eight interviews were transcribed for manual thematic analysis, with themes generated from coding of the data, and the coding process repeated in order to refine the themes as new data emerged (Saunders et al., 2012). Transcripts were emailed to participants for review and agreement, with one participant requesting minor changes. Thematic analysis was then independently undertaken by a second reviewer for coding in order to agree a consensus of the final themes and findings. All participants were sent a draft manuscript of the findings. The COREQ checklist was consulted to consider the quality of the methodology and reporting of the findings (Tong et al., 2007).

## Results

### Demographic data

The age of the participants ranged from 26 to 57, with three to 25 years of clinical experience, as shown in Tables 2 and 3. Five of the participants were male and three were female.

**Table 2.** Age range of participants.

Age range	Frequency
26–30	1
31–35	1
36–40	1
41–45	1
46–50	0
51–55	3
56–60	1

**Table 3.** Clinical years of experience of participants.

Years of experience	Frequency
0–5	3
6–10	2
11–15	1
16–20	1
21–25	1
26–30	0

### Thematic analysis

Three main themes were identified from analysis of the data relating to the ECHO programme. These were (1) programme structure, (2) factors influencing engagement and (3) professional impact. These are explored in further detail below, with supporting quotes provided. Some quotes were edited slightly to improve grammar only.

#### Programme structure

The format and structure of the programme provided access to a new experience for participants, with benefits derived from expert didactics and peer case studies, multi-professional participation and the frequency and length of each session.

The ECHO methodology was highly valued among the participants, as the facilitation was viewed as professional and allowed everyone the opportunity to contribute where required. The format of a short expert didactic presentation followed by participant case studies to learn and share practice encouraged clinicians to relate this back to their subsequent clinical care.

*I thought the presentation followed by the scenarios which allowed me to think about how it all fitted together was quite good and helped, then all I needed afterwards was to have the patient contact in my next shift and think it through in practice*. (P1)

Clinical expertise and subject relevance to the participant’s role and clinical practice were considered critical success factors for the programme, enabling clinicians to make the link between theory, reflective discussion and their own clinical practice. Relevant topics created an environment for debate in which expert speakers were highly regarded.

*The wealth of knowledge that came through from different professional backgrounds was good, the knowledge of the palliative care staff was really good and the general practitioner was also interesting.* (P3)

*I liked the guest speaker of each session, they were good because they were relevant to the topic which made it interesting, and they could answer all of our questions.* (P7)

The ability to cover a broader range of topics across regular short sessions provided the opportunity to undertake frequent education and supervision activities in a way not previously achieved. The difference between infrequent face-to-face sessions and a regular but short virtual community of practice offered a new way to develop skills and share practice.

*This is much better because with any other clinical updates you can go for quite a while without any training, but because of the short duration and higher frequency of the ECHO programme you can cover more subjects. People talk about ECHO now out on the road asking how to get onto it*. (P3)

However, the impact of shift work meant that planning the times of ECHO sessions required careful consideration in order to maximise participant attendance.

*For people who work shifts in our sort of environment the afternoon sessions would be better, because you’re not always guaranteed to finish your shift on time either. If you had been on night shift, having to get up for it [ECHO session] was a challenge, but it was worth getting up for*. (P3)

#### Factors influencing engagement

A number of factors were identified to increase participant engagement around the highly accessible and convenient nature of Project ECHO, the ability to network with peers and experts and the need for session preparation and time management.

Accessibility was viewed as one of Project ECHO’s greatest benefits, enabling smaller continuing professional development (CPD) sessions than previously available. Accessing ECHO sessions from home enabled a highly convenient mode of learning, especially for those who had busy schedules outside of working hours.

*I think for my personal situation and being tied with the kids it was a lot easier knowing I could do it from home, without having to commit to days out of the house and arranging childcare, so it definitely made it easier*. (P8)

Despite this, one participant recognised the commitment required in joining the planned sessions in order to create the community of practice.

*I think it’s much easier to do when it’s accessible like that. I can imagine that some people think you can quickly dip in and out, but you have to make it clear that you have to devote the time to get the best out of it*. (P8)

A positive effect of accessibility was the time and cost savings associated with travelling to venues for face-to-face sessions, this being recognised by the participants as a saving both personally and for the employing organisation.

*It was that nice small chunk that worked, whereas if I wanted to do this at [named venue] I’d have to be up early in the morning and beat the traffic and find parking, there’s an economic benefit here*. (P1)

Project ECHO provided the opportunity for paramedics to network with peers from across the region and with experts from other clinical disciplines, enabling the virtual community to meet on a regular basis.

*It’s not very often that we would have training with so many people who are at the forefront of their specialism, to have so many consultants and nurses all in one virtual room at the same time was amazing. The way it [ECHO] encouraged integration in our practice was good.* (P8)

*My experience of the whole programme was that it was the first time where I felt that it was a community of learning where paramedics were treated as experts in our own field.* (P8)

Areas for improvement were often related to the time management of the sessions, where disseminating presentations before each session would have allowed participants the opportunity to digest the content in advance. Moreover, the limited time left one clinician presenting a case study feeling rushed and unable to share his experiences well enough.

*I personally like having something to write on and look at later, and because everything was happening so quickly I couldn’t easily keep up. I’d have liked to have received the hand-outs before the session. (*P5)

*We were getting towards the end of the session and we were running out of time and I found that I didn’t get the chance to express myself properly. It was a really difficult case that I wanted to discuss . . . it was complex and because we were rushed we didn’t really get to do it justice.* (P1)

#### Professional impact

Project ECHO’s positive impact on professional practice was highlighted in a number of different ways. Through regular case studies and sharing of practice and experiences, participants were able to benchmark their own practice with others, allowing them to develop new capabilities and skills in a way that transfers to situations beyond end of life.

An important point raised by all participants was that the programme allowed them to benchmark their own clinical practice against the virtual community, enabling them to accept the complexity of EoLC.

*It was really great for me to hear other people talking about their thought processes on complex cases and how they think it through. It was the honesty about how it wasn’t black or white, right or wrong. To hear other people talking about that really helped me*. (P8)

Improved confidence and clinical competence were reported as a result of the programme, in terms of confidence to engage with the ECHO sessions and in clinical practice related to EoLC with technical and non-technical skills.

*I’ve been able to speak with families and patients about their future, having conversations about the next year or the next few months and raising the prospect of advance care planning and palliative care, I wouldn’t have dared to do that and broach the subject before the ECHO programme*. (P5)

A number of participants identified a ripple effect back in clinical practice in two distinct ways. Firstly, ECHO sessions were discussed back in the workplace with colleagues where the learning would be shared among the wider clinical team. Secondly, some participants felt that they had been able to apply new knowledge and skills to clinical presentations beyond end of life, particularly non-technical skills such as communication.

*I discussed ECHO a lot back on station including with my supervisor, and now he’s on the course and says it’s absolutely fantastic. When you’re on 999 calls and other clinicians ask why I’ve done something, I say it’s because I’ve been on ECHO. When I’m on station after doing ECHO I’d be discussing the content of my learning with colleagues, and I’d also be having those discussions right after cases too.* (P2)

*I can think of three cases since where the patients weren’t end of life but I’ve been able to have better conversations with them about their situation, what services are already involved, what kind of decisions they’ve made and thinking about whether hospital is in their best interests or not.* (P8)

## Discussion

This service evaluation set out to understand the experiences, benefits and challenges of an EoLC Project ECHO for ambulance clinicians in one English ambulance service, and any perceived impact upon their clinical practice.

Overall, Project ECHO was viewed positively by all participants interviewed. One of the distinguishing features of Project ECHO from other educational interventions was the ability to create a virtual community of practice, which for paramedics and other ambulance clinicians offered a new experience. Through the virtual community, our participants were able to regularly network with their peers and a range of other healthcare professionals, and valued the learning from this. This differs greatly from traditional CPD opportunities often limited to single educational events that prevent the creation of communities of practice.

For ambulance clinicians, the ability to work in groups and benchmark own practice is a challenge given the isolated nature of the role. Through expert didactics and peer case-based discussions, participants could benchmark their own practice which provided the opportunity for reflection and was reported to improve their confidence in clinical practice. This is consistent with the findings by Arora et al. (2010) that align Project ECHO with the educational theories of social cognitive and situational learning, allowing participants the opportunity to collaborate, learn from communities and implement new skills and behaviours in practice.

In this study, the concept of a ripple effect has provided new insights for the implications of Project ECHO, where participants discussed their learning with peers back in the workplace thus creating new learning opportunities for others. Moreover, the ripple effect was also described as a process where clinicians were able to apply new skills to situations beyond the subject of the programme they took part in. Creating novel educational opportunities that are highly accessible and of short durations yet much more frequently available may lead to this ripple effect occurring more often throughout clinical teams, with positive implications for clinical practice.

Consistent with implementation literature by Serhal et al. (2018), the success of the programme relies on fidelity to the ECHO methodology where our participants also found the format, subject relevance and frequency of sessions to be important. Challenges found in this study were related to time limitations, where some participants felt rushed in their contributions and favoured dissemination of presentation notes in advance. The concept of fidelity should be considered by organisations implementing ECHO given the investment required, and the need to avoid any programme failure associated with poor engagement.

While Zhou et al. (2016) acknowledge the preliminary evidence suggesting that Project ECHO is a potentially effective and cost-saving model, this was recognised as a benefit by our participants due to the accessible and convenient nature of the intervention, having further potential for ambulance services that cover large geographical areas. Furthermore, the COVID-19 pandemic presented significant challenges in education and CPD delivery, creating opportunities for Project ECHO to respond by delivering virtual communities of practice through digital technologies to clinicians who require enhanced supervision and support through uncertain times.

Despite the increased use of videoconferencing for meetings, education and some aspects of clinical practice, the use of Project ECHO as a methodology alongside these technological platforms should be considered as one option for enhancing education and supervision for remote clinical teams, as this represents a unique opportunity to connect clinicians and develop practice for patient benefit.

One limitation of this study is the small nature of the programme. Given that the pilot site was the first in the world for any ambulance service to the authors’ knowledge, further evaluation is required in other ambulance services and across wider clinical subjects to determine suitability for this professional environment. In addition, the interviews were designed and conducted by the lead author who was part of the ECHO delivery programme, resulting in potential bias in evaluation design and reporting.

## Conclusion

Our findings indicate that Project ECHO has potential utility in ambulance services where clinicians are spread across large geographical regions, improving access to education and developing new mechanisms of support through expert and peer sharing of practice. Ambulance services should consider implementing Project ECHO as part of formal education and CPD opportunities, while increasing access to supervision and peer review.

Further research is required to understand the practical implications across a broader range of subjects, its impact upon well-being and organisational belonging and any impact on patient outcomes.

## Author contributions

AH conceived the idea, designed and conducted the evaluation and wrote the manuscript. LM conceived the idea, delivered the ECHO sessions, provided senior clinical oversight and reviewed and revised the manuscript. JM worked with AH to analyse the data and in thematic analysis, and reviewed and revised the manuscript. PT assisted with interpretation of findings, supervised study conduct and revised drafting and editing of the manuscript. SK provided overall programme and study supervision, leads and implemented Project ECHO and reviewed the manuscript. AH acts as the guarantor for this article.

## Conflict of interest

None declared.

## Ethics

Formal ethics approval was not required as this was a service evaluation rather than research. However, the project was registered with the audit and service evaluation team at St. Luke’s Hospice Sheffield, to ensure that ethical procedures were followed. The attendees’ employing organisation supported the programme. The participants signed consent and GDPR forms before attending the education sessions for the first time, and written consent was also gained for participation in the interviews.

## Funding

None.
